# Non-invasive detection of intracranial hypertension using a simplified intracranial hemo- and hydro-dynamics model

**DOI:** 10.1186/s12938-015-0051-3

**Published:** 2015-05-30

**Authors:** Kwang Jin Lee, Chanki Park, Jooyoung Oh, Boreom Lee

**Affiliations:** Department of Medical System Engineering (DMSE), Gwangju Institute of Science and Technology (GIST), Gwangju, South Korea; School of Mechatronics, Gwangju Institute of Science and Technology (GIST), Gwangju, South Korea

**Keywords:** Intracranial pressure, Pulsatility index, Intracranial hemo- and hydro-dynamics model, Cerebral blood flow velocity, Intracranial hypertension, Valsalva maneuver

## Abstract

**Background:**

Monitoring of intracranial pressure (ICP) is highly important for detecting abnormal brain conditions such as intracranial hemorrhage, cerebral edema, or brain tumor. Until now, the monitoring of ICP requires an invasive method which has many disadvantages including the risk of infections, hemorrhage, or brain herniation. Therefore, many non-invasive methods have been proposed for estimating ICP. However, these methods are still insufficient to estimate sudden increases in ICP.

**Methods:**

We proposed a simplified intracranial hemo- and hydro-dynamics model that consisted of two simple resistance circuits. From this proposed model, we designed an ICP estimation algorithm to trace ICP changes. First, we performed a simulation based on the original Ursino model with the real arterial blood pressure to investigate our proposed approach. We subsequently applied it to experimental data that were measured during the Valsalva maneuver (VM) and resting state, respectively.

**Results:**

Simulation result revealed a small root mean square error (RMSE) between the estimated ICP by our approach and the reference ICP derived from the original Ursino model. Compared to the pulsatility index (PI) based approach and Kashif’s model, our proposed method showed more statistically significant difference between VM and resting state.

**Conclusion:**

Our proposed method successfully tracked sudden ICP increases. Therefore, our method may serve as a suitable tool for non-invasive ICP monitoring.

## Background

Intracranial pressure (ICP) is the pressure inside the cranium that exerts on the neural tissue and cerebrospinal fluid (CSF). Normal range of mean ICP for a healthy subject in supine position is 5–15 mmHg [1]. However, ICP can be elevated remarkably due to variety of intracranial pathologies such as intracranial hemorrhage, cerebral edema, brain tumor, etc. According to the Monro–Kelli hypothesis, as ICP increases, cerebral perfusion pressure correspondingly decreases [2]. Therefore, increased ICP may cause ischemic stroke, neural damage, and even brain death. For this reason, monitoring of ICP is essential for preventing secondary brain damage. Currently, ICP monitoring is frequently considered for patients with severe brain injuries, cerebrovascular accidents, hydrocephalus, hypoxic brain injuries, central nervous system infections, and fulminant hepatic failures [3–5].

For direct ICP monitoring, an invasive surgical procedure involving drilling a burr hole in the skull for injection of a catheter is necessary. Therefore, invasive ICP monitoring methods have many disadvantages including the risk of infections and hemorrhage [6], high-cost of surgical procedures, and the need for a trained neurosurgeon. Since many limitations of invasive ICP monitoring can make measuring the ICP difficult, non-invasive methods have been proposed to estimate ICP using related physiological variables [7–9]. However, previously proposed methods do not provide sufficiently accurate ICP values. Thus, they are not appropriate tools for routine general clinical use.

Recently, many researchers frequently used pulsatility index (PI) for estimating ICP non-invasively [10–12]. Measured cerebral blood flow velocity (CBFv) from transcranial Doppler (TCD) is known to be strongly related to ICP [11]. The PI can be calculated easily using the CBFv. The PI derived from CBFv is a useful indicator for identifying patients with raised ICP. Moreover, the mean deviation between the PI-based ICP value and invasively measured ICP was 4.2 mmHg [13], indicating that PI based approach is clinically acceptable. However, PI based approach did not consider various factors except CBFv. Therefore, if the sudden increase in ICP does not affect the waveform of CBFv, we are unable to detect a sudden and developing intracranial hypertension [14]. Some non-invasive ICP methods used the morphological clustering algorithms to estimate the ICP value from TCD measurements of CBFv waveform [15, 16]. To generate the ICP estimate, the kernel regression mapping which is derived from morphological features from TCD based CBFv is necessary [16]. However, this method could not explain adequately the underlying mechanistic model. Furthermore, to get more accurate ICP estimates, the morphological approach needs many number of training set on a reference population.

On the other hand, many cerebral hemodynamics models, adaptable to non-invasive ICP estimation, have been reported so far. Ursino proposed hemo- and hydro-dynamic models about CSF fluid dynamics in 1988 [17, 18]. Later, the reduced Ursino model was reported using a simpler mathematical model [19]. This model identifies interactions among ICP, cerebral blood volume, and auto-regulation. Especially, it has been shown to be efficient in providing cerebral auto-regulation. However, this model still has a mathematically complex formula, which involves fitting model parameters such as the bridging vein resistance, and intracranial elastic coefficient.

Recently, Kashif et al. proposed a very simple hemodynamics model for non-invasive ICP monitoring by using the CBFv and arterial blood pressure (ABP) [20]. Their model-based approximation of the ICP estimated some model parameters including intracranial resistance, and elastic coefficient through a physiological model of cerebrovascular dynamics. Kashif method seemed to be successful in tracking ICP changes. Nonetheless, this estimation algorithm may be unsuitable to respond to sudden increases in ICP because of the very long time window (60 s).

In the current paper, we will propose a simplified intracranial hemo- and hydro-dynamics model for the detection of intracranial hypertension in real time. Our method does not need to estimate compliance parameters but only considers resistance parameters, thereby establishing a simpler intracranial hemo- and hydro-dynamics model compared to existing other models. Using our proposed model, we designed an ICP estimator with the direct current (DC) trends of ABP and CBFv as inputs. To verify the proposed method, we constructed the simulation cerebral blood flow (CBF) signal using the original Ursino model with a real ABP. We subsequently estimated the ICP using our proposed method from the ABP and simulation CBF. Finally, our method and two non-invasive ICP estimation approaches were applied to experimental data which consisted of ABP and CBFv. To assess the performances of the methods, we conducted test for the detection of sudden ICP change caused by Valsalva maneuver (VM). Since VM raises intrathoracic pressure which significantly affects systemic and cerebral circulation, ICP also suddenly increases during VM [21]. Therefore, VM phase can be regarded as intracranial hypertension state. Moreover, we compared the ICP estimation performance of our method with that of the PI method and Kashif method. To identify the discriminability about sudden increases in ICP during VM (intracranial hypertension state), statistical test was performed. We will show that our method has better detecting performance about sudden change of ICP than the PI method and Kashif method.

## Methods

### Pulsatility index based approach

Despite the close ICP estimation using the PI, it is unable to detect the absolute ICP level. PI based on the Gosling formula, and conventionally applied to a single beat, can also be used to monitor beat-to-beat changes. Given one pulse, PI is as follows:

1 $${\text{PI}} = \, ({\text{CBFv}}_{\text{systolic}} - {\text{CBFv}}_{\text{diastolic}} )/{\text{CBFv}}_{\text{mean}}$$

where CBFv_systolic_, CBFv_diastolic_, and CBFv_mean_ represent the peak systolic velocity, the end-diastolic velocity, and the mean velocity for CBF, respectively. Since the PI is derived from the waveform of one pulse, the PI must be updated at the next pulse. The ICP can be estimated by a linear regression of the PI [11]. In order to estimate the ICP from the PI, we adopt the linear regression equations as follows,

2 $${\text{ICP}} = {\text{a}} \times {\text{PI}} + {\text{b}}$$

where, a and b represent the regression coefficients. We utilized the values of the previous study [12] as the regression coefficients of Eq. (2). Additionally, we added some offset value to the regression coefficients in order to compensate for the gap between the estimated value and normal ranges of ICP (5–15 mmHg). As a result, 5.305 and 4 were assigned to regression coefficients a and b, respectively.

In this study, we will refer to the ICP estimation approach based on the linear regression of the PI as the “PI method”. In order to compare the performance of the PI method with that of our method, the estimated ICP was utilized.

### Kashif method

**Figure 1:**
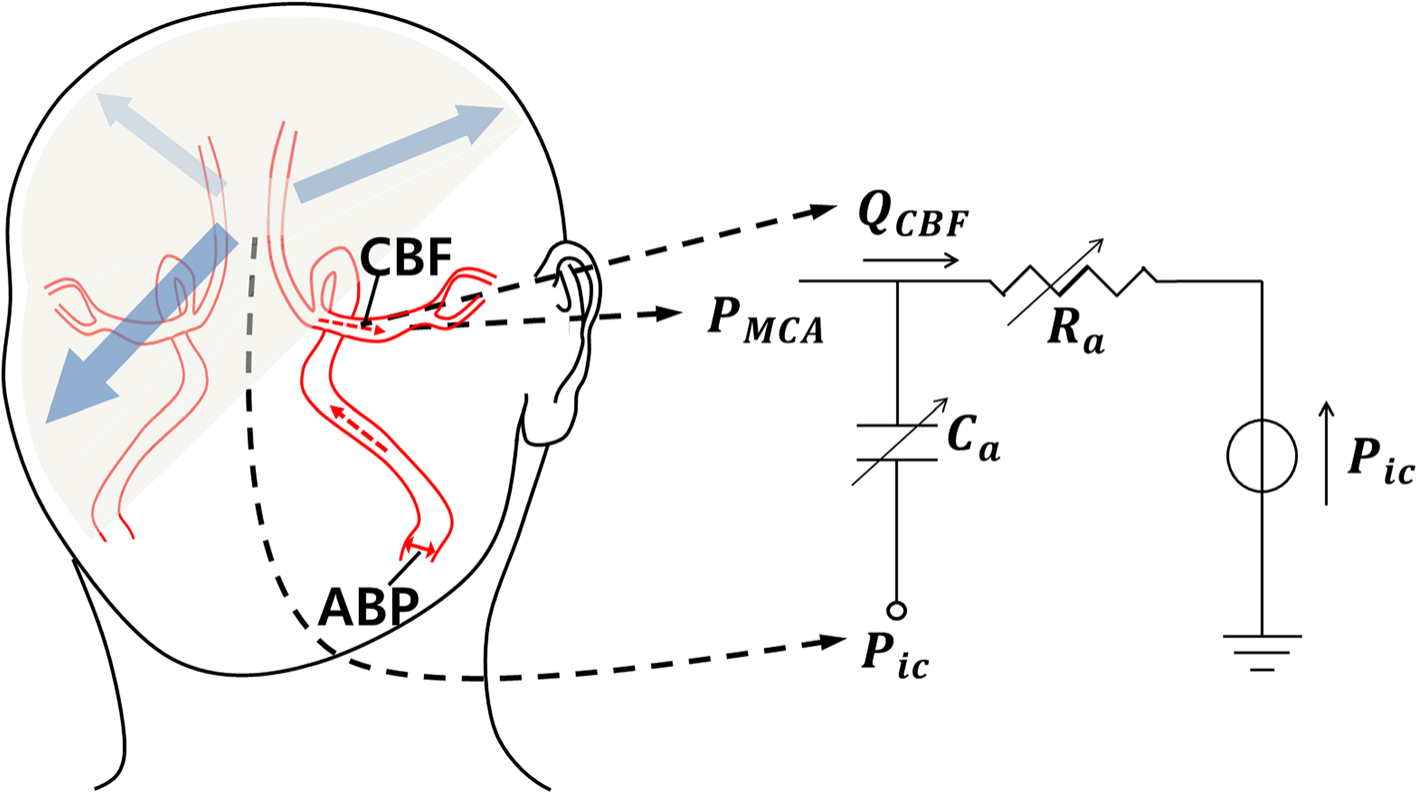
Kashif’s dynamic model.

Since radial ABP and ABP at middle cerebral artery (MCA) have different systolic upstroke time, an additional time-shift is needed for using the Kashif’s simplified model (see Figure 1). To find the time-shift τ, one should detect peaks of CBFv and ABP waveform within each beat. ABP at MCA can obtain as follow:

3 $${\text{P}}_{\text{MCA}} \left( {\text{t}} \right) = {\text{P}}_{\text{r}} \left( {{\text{t}} + \, \tau } \right)$$

where P_MCA_ is the middle cerebral artery pressure, P_r_ is radial ABP, and τ is the time-shift.

Figure 1 illustrates the hemodynamics circuit of Kashif’s simplified model. Q_CBF_ is CBFv and P_ic_ is ICP. The algorithm is assumed to be constant at mean ICP value over the estimation window. From this assumption, Q_CBF_ can be expressed with parameters R_a_ and C_a_ which represent resistance and compliance of intracranial artery:

4 $${\text{Q}}_{CBF} = \frac{{P_{MCA} - P_{ic} }}{{R_{a} }} + C_{a} \frac{{dP_{MCA} }}{dt}$$

To estimate ICP value, the algorithm follows two steps: First, the compliance parameter C_a_ is estimated, and then, the estimate of C_a_ is used to estimate R_a_ in the Kashif’s simplified model. The second step is the estimation of ICP. The ICP estimation algorithm with Kashif’s simplified model is briefly described as follows.

1. ABP and CBFv are annotated for beat onsets. Here CBFv is assumed to be proportional to CBF [20].
2. Parameters C_a_ and R_a_ are estimated within each one beat. Since most Q_CBF_ pass the compliance branch during the sharp transitions in P_MCA_, the model can be simplified to a capacitor-only branch. Thus parameter C_a_ can be estimated. After obtaining the estimate of C_a_, the estimate of CBF ${\hat{\text{Q}}}_{\text{CBF}}$ is obtained, and then we can estimate parameter R_a_ using two time-instants of ABP and ${\hat{\text{Q}}}_{\text{CBF}}$. Estimation process of two parameters C_a_ and R_a_ are detailed in [20].
3. The estimate of R_a_ is then substituted into the following Eq. (5), and an ICP estimate can be obtained for the given one cycle.

5 $$P_{ic} = P_{MCA} - R_{a} \hat{Q}_{CBF}$$

### Proposed method: simple resistance model and simple resistance method

Many mathematical models have been utilized to estimate hidden health states such as ABP and ICP. Specifically, Ursino’s hemo- and hydro-dynamics model was the cornerstone of the model-based ICP estimation field [18]. In this section, we will present a novel model, which was developed as a modification of the original Ursino model [18] and an ICP estimation algorithm based on the novel model. For the sake of simplicity, we will refer to the simplified model as the “SR model” (simple resistance model) and our ICP estimation algorithm as the “SR method”.

**Figure 2:**
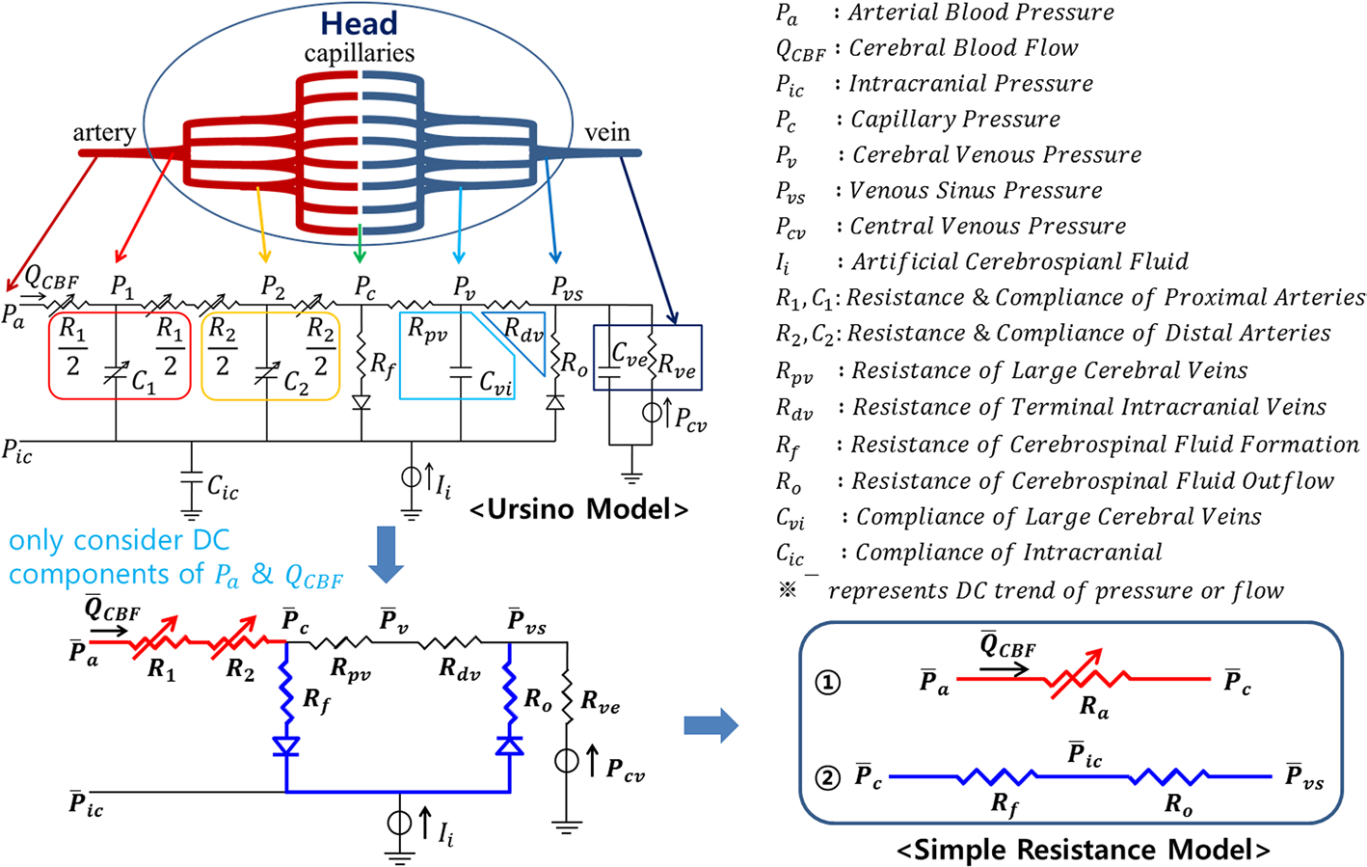
Simplification steps from the original Ursino model to the SR. First, we only considered the DC components of the arterial blood pressure (P_a_) and cerebral blood flow (Q_CBF_) in order to eliminate the compliance term. Second, we extracted two resistance circuits and referred to set of these two circuits as the “SR model” (simple resistance model).

The SR model is derived from the original Ursino model [18]. Figure 2 shows the simplification steps. First, we only consider the DC component of the ABP, which is the input of the original Ursino model. We can ignore the compliances in the original Ursino model, because the compliances have infinite impedance when the input is DC. From this step, we can get the resistance model without compliances. Next, we extract two simple circuits from the resistance model and we refer to them as the “SR model”. The first circuit of the SR model is the intracranial hemodynamics model extending from the intracranial arteries to the capillaries, and the other circuit represents the intracranial hydrodynamics model.

**Figure 3:**
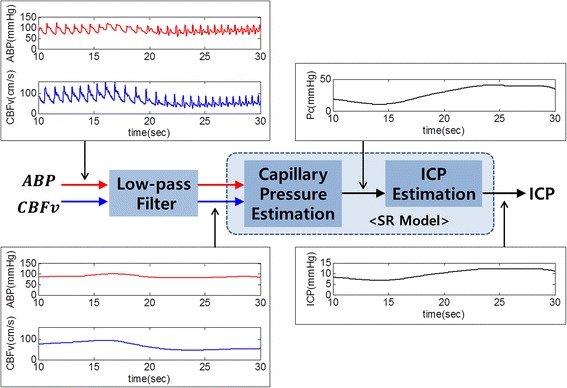
The SR method for estimating the ICP using the ABP and CBFv as input. The ABP and CBFv applied low-pass filter and the estimated ICP was obtained from the SR model, which consists of two simple circuits. One is used to estimate the capillary pressure while the other is utilized for estimating ICP.

Based on the SR model, the SR method was designed to estimate the ICP through a series of three processes (see Figure 3). The first process involves low pass filtering (0.2 Hz). Since the SR model considers only DC components, it is necessary to suppress the alternating current (AC) components of the ABP and CBFv. The next process involves capillary pressure estimation from the ABP and the CBF by using the first circuit of the SR model as follows:

6 $${\text{P}}_{\text{c}} = {\text{ P}}_{\text{a}} - {\text{R}}_{\text{a}} \cdot {\text{Q}}_{\text{CBF}}$$

where, P_c_ and P_a_ represent the capillary pressure and ABP, respectively. R_a_ represents the resistance of intracranial arteries and the Q_CBF_ indicates the CBF. In the last step, the ICP is calculated from the estimated capillary pressure and the second circuit of the SR model as follows:

7 $${\text{P}}_{\text{ic}} = {\text{ P}}_{\text{vs}} + \left( {{\text{P}}_{\text{c}} - {\text{P}}_{\text{vs}} } \right){\text{R}}_{\text{o}} /\left( {{\text{R}}_{\text{f}} + {\text{R}}_{\text{o}} } \right)$$

where, P_ic_ and P_vs_ represent the ICP and venous sinus pressure, respectively. R_f_ and R_o_ represent resistances of CSF formation and outflow, respectively. Equation (7) has the same meaning of the linear interpolation between the P_c_ and P_vs_. We set the values of R_a_, P_vs_, R_f_, and R_o_ according to one reference [18, 19]: R_a_ = 6 mmHg s/ml, P_vs_ = 6 mmHg, R_f_ = 2,380 mmHg s/ml, and R_o_ = 523 mmHg s/ml.

One of the input data of the SR method is the CBF. Although it is difficult to measure CBF directly, the CBFv is approximately proportional to CBF [18]. Hence, we calculate the CBF by the proportional value of the CBFv as follows,

8 $${\text{Q}}_{\text{CBF}} = {\text{CBFv}} \times \alpha$$

where α represents the proportional factor. We chose 0.15 as the value of α because CBF is about 11.67 ml/s and CBFv is usually from 70 to 80 cm/s for healthy people [22]. As a result, ICP can be simply estimated from low pass filtered ABP and CBFv using the SR method. The total process is depicted on Figure 3.

### Data

**Figure 4:**
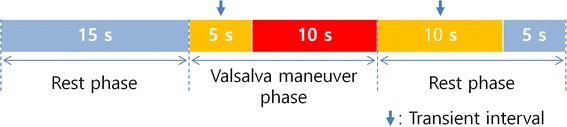
Experiment paradigm. During the first 15 s, each participant takes a rest. Next, participants perform the VM for 15 s. The participants then take a rest again for 15 s.

Since previous study already showed that peripheral and central mean ABPs was interchangeable [23], the SR method utilized DC trend of peripheral ABP from the index finger as the input. Furthermore, some methods also used non-invasively measured peripheral ABP for estimating ICP [16, 20]. Therefore, as the inputs of our method, ABP was measured from the left hand index finger using the NIBP100 (Biopac Systems, Inc., Santa Barbara, CA, USA). The system employed a tonometric technique that has been previously validated, which measured intra-arterial pulse pressure [24]. CBFv was measured at the MCA via EZ-Dop^®^ (DWL, Compumedics Germany GmbH, Singen, Germany) TCD ultrasound device. The waveform of ABP and CBFv are simultaneously collected by MP100 system (Biopac Systems, Inc., Santa Barbara, CA, USA) with 1 k Hz sampling frequency, respectively. Since it was known that ICP level was increased by the effect of a VM [21], the ICP tracking performance could be assessed by detecting a VM (intracranial hypertension state) when a subject alternately takes a rest and a VM. Figure 4 represents the total 45-s experimental protocol. A subject took a rest during the first 15 s and then performed a VM during the next 15 s. After that, the subject had a rest for 15 s. To remove the uncertain states in each trial, we did not use the initial 5 s of VM phase and 10 s of later rest phase as indicated in Figure 4. Eight healthy people (age, 29 ± 4; weight, 63.5 ± 9 kg) participated in this experiment and each subject performed 20 trials in total. We informed the participants about the experimental procedures and potential risks. Written informed consents were obtained from all the subjects. The institutional review board (IRB) of the Gwangju Institute of Science and Technology approved all procedures and protocols in this study.

## Results

### Simulation

**Figure 5:**
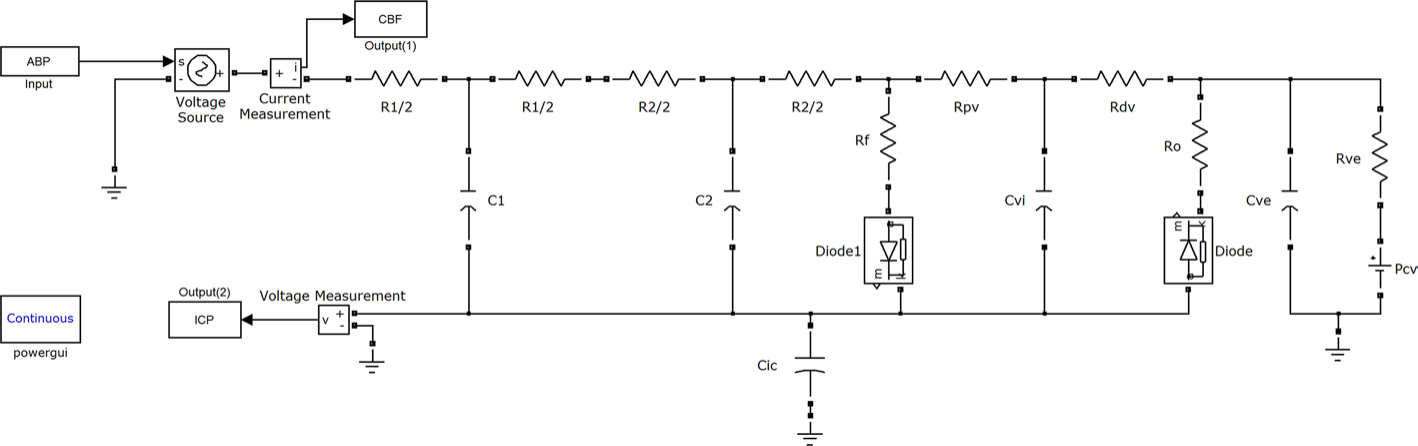
Simulation circuit for the MATLAB^®^ Simulink. The original Ursino model was used for simulation.

**Figure 6:**
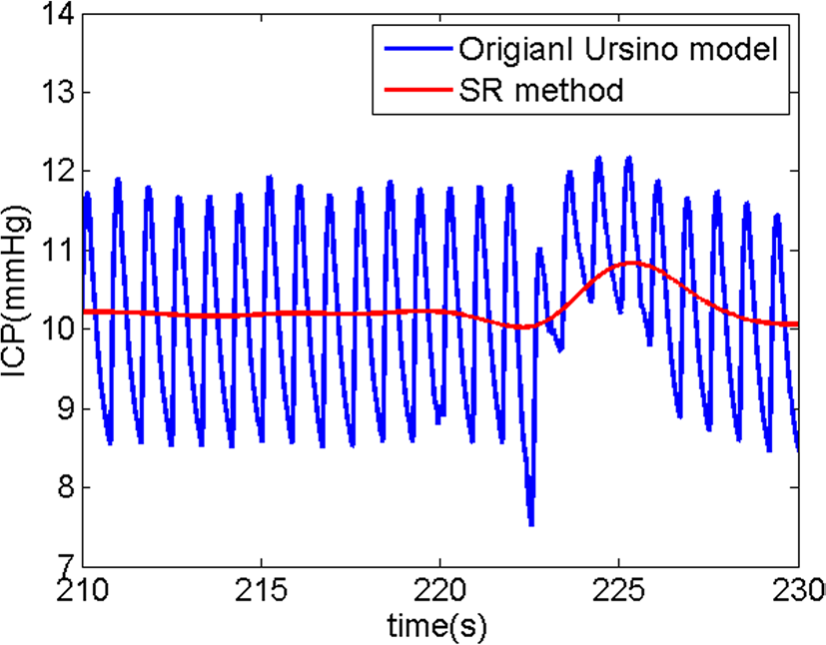
Simulation result. ICP was estimated from the real ABP and the simulation CBF with the original Ursino model. The *blue line* indicates the ICP simulated by the original Ursino model while the *red line* indicates the estimated ICP via the SR method.

In order to verify the validity of the SR model and method, we performed a simulation based on the original Ursino model in MATLAB^®^ Simulink. As can be seen in Figure 5, we simulated the equivalent circuit for the original Ursino model and utilized the real ABP data of the PhysioNet database [25] as the input of the circuit. We obtained simulated CBF and ICP data from the computer simulation. With the given real ABP and simulation CBF, we estimated ICP using the SR method, and it was compared with the simulation ICP (see Figure 6). We calculated root mean square errors (RMSEs) between the estimated ICP and the simulation ICP for twenty data sets. The mean of RMSE for all simulation data was 1.36 mmHg with a standard deviation of 0.2 mmHg. That is, even though the SR model greatly simplifies the original Ursino model, the SR method shows high tracking performance for the ICP generated from the original Ursino model. For reference, model parameters of the original Ursino model are set to the appropriate values explained in [18, 19] in detail (see Table 1).

**Table 1:** Parameters of the original Ursino model for simulation

Symbol	Name	Value
R_1_	Resistance of proximal arteries	2.4 mmHg s/ml
C_1_	Compliance of proximal arteries	0.03 ml/mmHg
R_2_	Resistance of distal arteries	3.64 mmHg s/ml
C_2_	Compliance of distal arteries	0.06 ml/mmHg
R_f_	CSF formation resistance	2,380 mmHg s/ml
R_o_	CSF outflow resistance	526 mmHg s/ml
R_pv_	Proximal venous resistance	0.88 mmHg s/ml
C_vi_	Proximal venous compliance	0.46 ml/mmHg
R_dv_	Bridging vein resistance	0.614 mmHg s/ml
R_ve_	Extracranial venous resistance	0.16 mmHg s/ml
C_ve_	Extracranial venous compliance	2.34 ml/mmHg
P_cv_	Central venous pressure	4 mmHg
C_ic_	Intracranial compliance	0.95 ml/mmHg

### Human subject experiment

**Figure 7:**
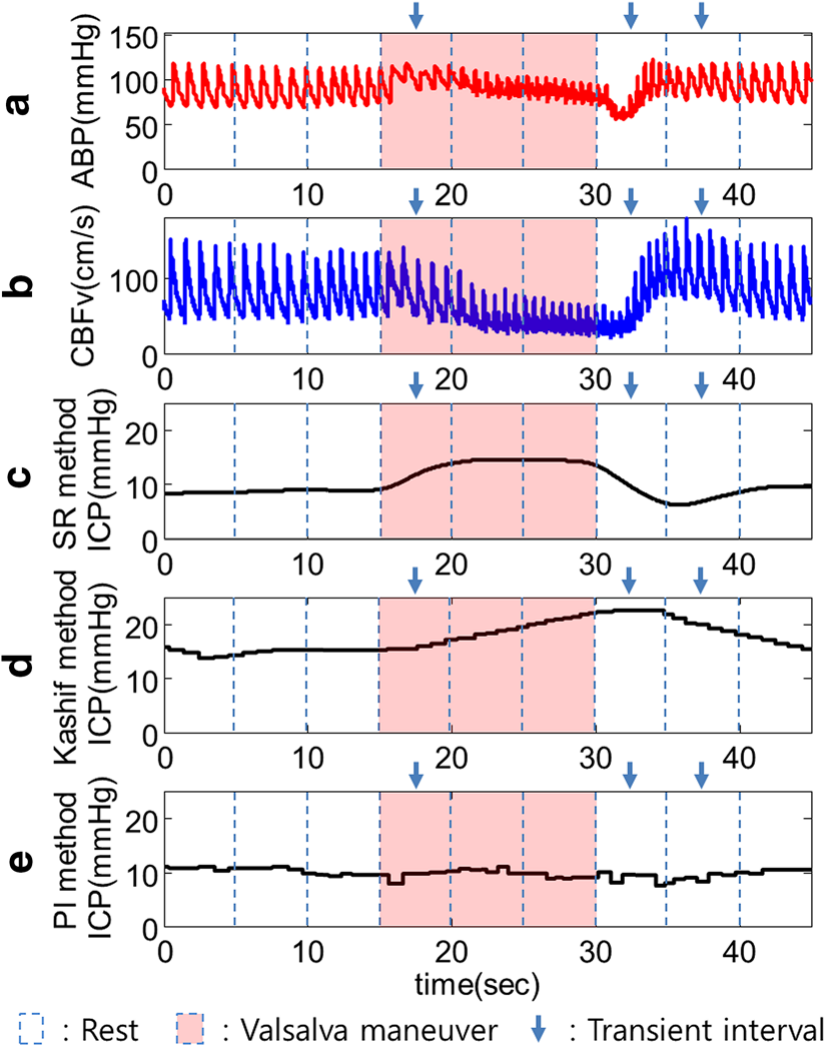
Estimated ICP level from measured ABP and CBFv. **a** Measured ABP signal from the left index finger. **b** CBFv signal measured from the left MCA. **c** Estimated ICP using the SR method. This method can track higher ICP levels increased by VM. **d** Estimated ICP with Kashif method. It can react to the ICP elevation but its estimate is lagged. **e** Estimated ICP signal via PI. PI cannot track sudden ICP elevations during the VM phase.

**Figure 8:**
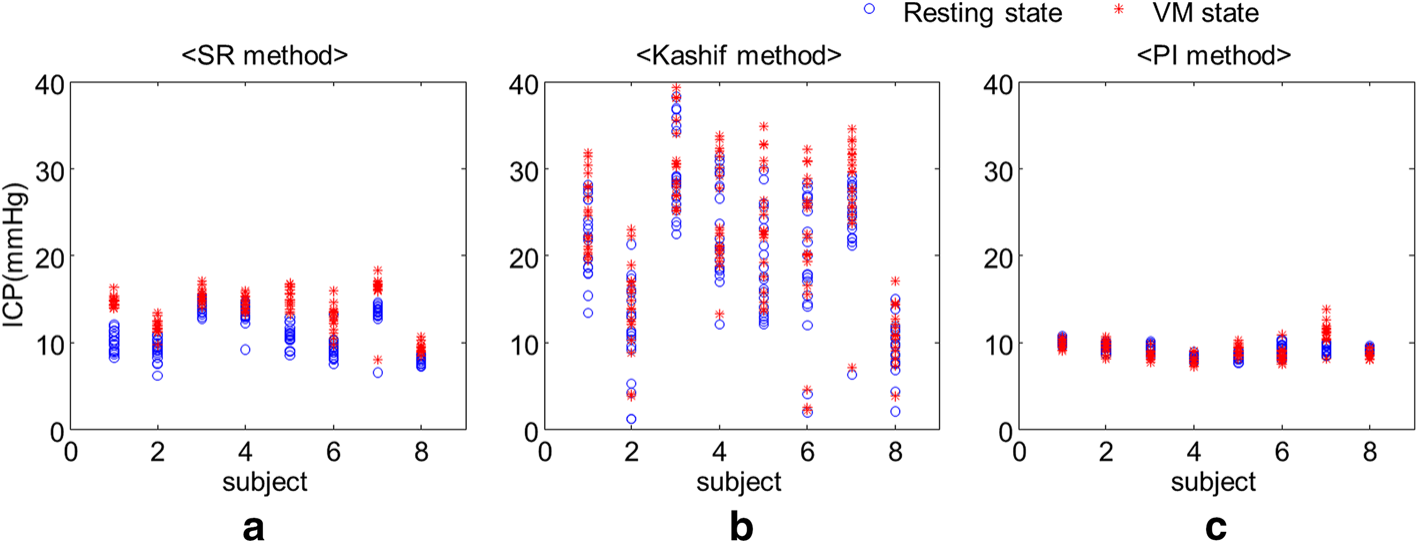
The mean values of ICP with respect to resting state and VM state for eight subjects. **a**, **b**, and **c** represent the mean values of ICP estimates for the SR method, the Kashif method, and the PI method, respectively. The *asterisk* and *circle marks* indicate resting and VM state.

In order to evaluate the SR method using human experimental data, we conducted the non-invasive detection of the intracranial hypertension phase using ABP measured from the left index finger and CBFv from the left MCA. The results of the non-invasively estimated ICP are shown in Figure 7. The third row (c) in Figure 7 reveals how our method can rapidly track increased ICP by VM. The fourth row (d) depicts the performance of Kashif method. It can react to the ICP elevation but its estimate is lagged. On the other hand, the bottom row (e) indicates that the PI cannot reflect the change at the VM phase well. Moreover, we computed the mean ICP value for resting and VM state using estimated ICP at each trial. The mean values for all trials are shown in Figure 8 and we can see the difference of distribution among the SR method, the Kashif method, and the PI method. Table 2 shows the mean and standard deviation values for resting and VM state. To verify the detection performance for ICP elevation, the one-tailed paired *t* test was used between resting and VM state for each subject. When the result show the significant difference (p < 0.05) between two phases for each subject, it is marked on Table 2 with an asterisk. As a result, the PI method could not estimate ICP increase at VM state and the Kashif method was insufficient, while the SR method well tracked sudden increases in ICP at VM state.

**Table 2:** Comparison of estimated ICP from the SR, Kashif method and the PI method for resting state and VM state (mean ± standard deviation, mmHg)

	SR method		Kashif method		PI method	
	Resting state	VM state	Resting state	VM state	Resting state	VM state
Subject 1	10.1 ± 1.1	14.7 ± 0.6*	21.6 ± 4.2	24.5 ± 4.1*	10.1 ± 0.3	9.8 ± 0.5
Subject 2	9.1 ± 1.1	11.8 ± 0.8*	11.3 ± 5.4	13.7 ± 5.0	9.5 ± 0.4	9.5 ± 0.7
Subject 3	14.1 ± 0.8	15.3 ± 0.8*	29.6 ± 4.9	31.2 ± 5.0	9.4 ± 0.4	8.4 ± 0.5
Subject 4	13.5 ± 0.8	14.7 ± 0.8*	23.5 ± 5.1	25.1 ± 5.4	8.2 ± 0.3	7.8 ± 0.4
Subject 5	10.6 ± 1.2	15.2 ± 1.1*	18.2 ± 5.8	22.7 ± 6.8*	8.3 ± 0.4	9.1 ± 0.5*
Subject 6	9.1 ± 0.9	12.6 ± 1.2*	19.3 ± 7.6	21.1 ± 9.1	9.2 ± 0.6	8.3 ± 0.5
Subject 7	13.5 ± 0.5	16.5 ± 0.5*	24.7 ± 2.8	28.7 ± 3.6*	9.1 ± 0.5	11.3 ± 0.8*
Subject 8	7.8 ± 0.5	9.3 ± 0.7*	9.1 ± 3.0	11.0 ± 3.2*	9.2 ± 0.2	8.5 ± 0.4
Total	10.9 ± 2.4	13.8 ± 2.4	19.7 ± 4.8	22.3 ± 5.3	9.1 ± 0.6	9.1 ± 1.1

* Represents p < 0.05.

## Discussion

For estimating the ICP, we proposed the SR model consisting of two simple circuits utilizing intracranial hemo- and hydro-dynamics that tracks ICP changes accurately using two input signals (CBFv and ABP). The SR method was verified by simulation using the PhysioBank data [25]. Since the data did not include measured CBFv data, we generated simulation CBF and ICP signal using the original Ursino model, and then used simulation CBF and real ABP as input data for the SR method. To assess the performance of our proposed method, we used RMSE between estimated ICP and simulation ICP generated by the original Ursino model. The SR method showed a mean RMSE of 1.36 mmHg, suggesting that this method can track ICP variation accurately. Although, the SR method is more simplified model than the original Ursino model, its ICP estimate was not significantly different with that of original Ursino model.

PI method has been widely used to estimate ICP noninvasively [10–12]. However, in this study, the limitation of PI was shown in terms of the ability of VM detection using human subject data. Real ABP and CBFv waveforms tend to rapidly change with VM. We assumed that real ICP would suddenly increase during the VM phase [21]. We confirmed that the estimated ICP using the SR method was elevated during the VM phase. Moreover, we showed the performance of detecting ICP changes using the statistical test. The SR method showed statistically significant differences between resting and VM state. However PI method could not show the differences because ICP elevation was not estimated during the VM phase. In other words, the SR method could successfully track ICP on the VM phase (intracranial hypertension) compared to the PI method. This is because PI was derived only from CBFv. If ICP was suddenly increased, CBFv could not always reflect ICP because higher ICP was also associated with several other physiological conditions such as ABP or PCO_2_ [26, 27]. Taken together, the PI method could not track the rapid change in ICP during intracranial hypertension caused by VM.

Recently, Kashif et al. also proposed new simplified model for estimating ICP [20]. In our human subject experiment, the Kashif method showed significant difference only for half of the subjects. This result suggested that the Kashif method could show better performance of ICP tracking than the PI method. However, their model estimates both resistance and compliance terms from the CBFv and ABP morphology, within the window. If CBFv and ABP are changing the level abruptly, it will be difficult to estimate these terms. Furthermore, since they calculate the ICP within a long window (60 s), their method is not appropriate for detecting abrupt ICP changes. On the other hand, as shown in experiment results, the SR method could detect sudden increases in ICP by VM. Furthermore, because the SR method only considers the DC components and the resistance term without compliance term, it has strong points not only for the robustness to the motion artifacts, but also in the design of the adaptive algorithm for the model parameters (R_a_ and C_a_ in Figure 1) that are regulated by cerebral autoregulation mechanisms.

Our investigation was to assess the feasibility of the SR method. However, in this study, we did not evaluate the SR method using the invasively measured ICP. Despite of this limitation, as shown in the results, the SR method could show sufficient performance to respond to abrupt changes in ICP immediately compared to other methods. In the near future, we have a plan to improve our proposed method using the invasively measured ICP in cooperation with a general hospital of Korea.

## Conclusions

In this study, we proposed a simple hemo-and hydro-dynamics model (SR method) to estimate ICP non-invasively. We showed the SR method could successfully track sudden increases in ICP compared to two other methods (the PI method and the Kashif method). Accordingly, the present study suggests that our proposed method may be a great tool for monitoring rapid changes in ICP non-invasively and it would help patients with regulating ICP within the appropriate range.

## Abbreviations

ICP: intracranial pressure
CSF: cerebrospinal fluid
CBF: cerebral blood flow
CBFv: cerebral blood flow velocity
ABP: arterial blood pressure
PI: pulsatility index
TCD: transcranial Doppler
VM: Valsalva maneuver
SR: simple resistance
DC: direct current
AC: alternating current
IRB: institutional review board
